# SIRT7 in the aging process

**DOI:** 10.1007/s00018-022-04342-x

**Published:** 2022-05-18

**Authors:** Francisco Alejandro Lagunas-Rangel

**Affiliations:** Department of Surgical Sciences, Uppsala University, BMC, Husargatan 3, Box 593, 751 24 Uppsala, Sweden

**Keywords:** Sirtuins, Hallmarks of aging, Ribosomal RNA, Nucleolus, Aging-associated diseases

## Abstract

Aging is the result of the accumulation of a wide variety of molecular and cellular damage over time. This has been associated with a number of features termed hallmarks of aging, including genomic instability, loss of proteostasis, telomere attrition, dysregulated nutrient sensing, mitochondrial dysfunction, cellular senescence, stem cell exhaustion, and impaired intercellular communication. On the other hand, sirtuins are enzymes with an important role in aging and life extension, of which humans have seven paralogs (SIRT1 to SIRT7). SIRT7 is the least studied sirtuin to date, but it has been reported to serve important functions, such as promoting ribosomal RNA expression, aiding in DNA damage repair, and regulating chromatin compaction. Several studies have established a close relationship between SIRT7 and age-related processes, but knowledge in this area is still scarce. Therefore, the purpose of this review was to analyze how SIRT7 is associated with each of the hallmarks of aging, as well as with some of age-associated diseases, such as cardiovascular diseases, obesity, osteoporosis, and cancer.

## Introduction

The sirtuin family of proteins is highly conserved, both functionally and structurally. Its members are found in most forms of life, including eubacteria, archaea, and eukaryotes [1, 2]. Mammals have seven sirtuins (SIRT1 to SIRT7) which are critical for regulating cell growth, energy metabolism, stress resistance, inflammation, circadian rhythms, neuronal function and aging [3, 4]. Overall, sirtuins are considered deacetylases, but it has been seen that they can also carry out other types of reactions such as adenosine 5′-diphosphate (ADP)-ribosylation, desuccinylation and demalonylation, among others [5]. Notably, sirtuin activities depend on the presence of nicotinamide adenine dinucleotide in its oxidized form (NAD+), which acts as a co-substrate and links sirtuins with cellular energy metabolism and nutritional intake [6]. Although sirtuins have many targets, some of the most important are histones because it is through them that these proteins can regulate gene expression. Indeed, sirtuins are considered within the class III histone deacetylase (HDAC) family of enzymes [7]. Based on phylogenetic analyses, the sirtuin protein family has been divided into five different classes, where SIRT1, SIRT2, and SIRT3 belong to class I, SIRT4 represents class II, SIRT5 belongs to class III, while SIRT6 and SIRT7 are class IV members [8]. SIRT7 is apparently only present in eukaryotes and appears to have been derived from a duplication of the SIRT6 gene that occurred during evolution at the *Coelomata* level, as other more primitive eukaryotes, including protozoa, *Pseudocoelomata*, and *Acoelomata*, lack SIRT7 genes [9]. SIRT7 is the least studied of the sirtuins, but recent advances have shown that it is also involved in numerous cellular processes, and its biological role is very important in health and disease [10]. Thus, the purpose of this review was to analyze the different functions of SIRT7 with special emphasis on describing the molecular mechanisms that link it to the aging process and its associated diseases.

## Brief introduction of SIRT7

**Fig. 1:**
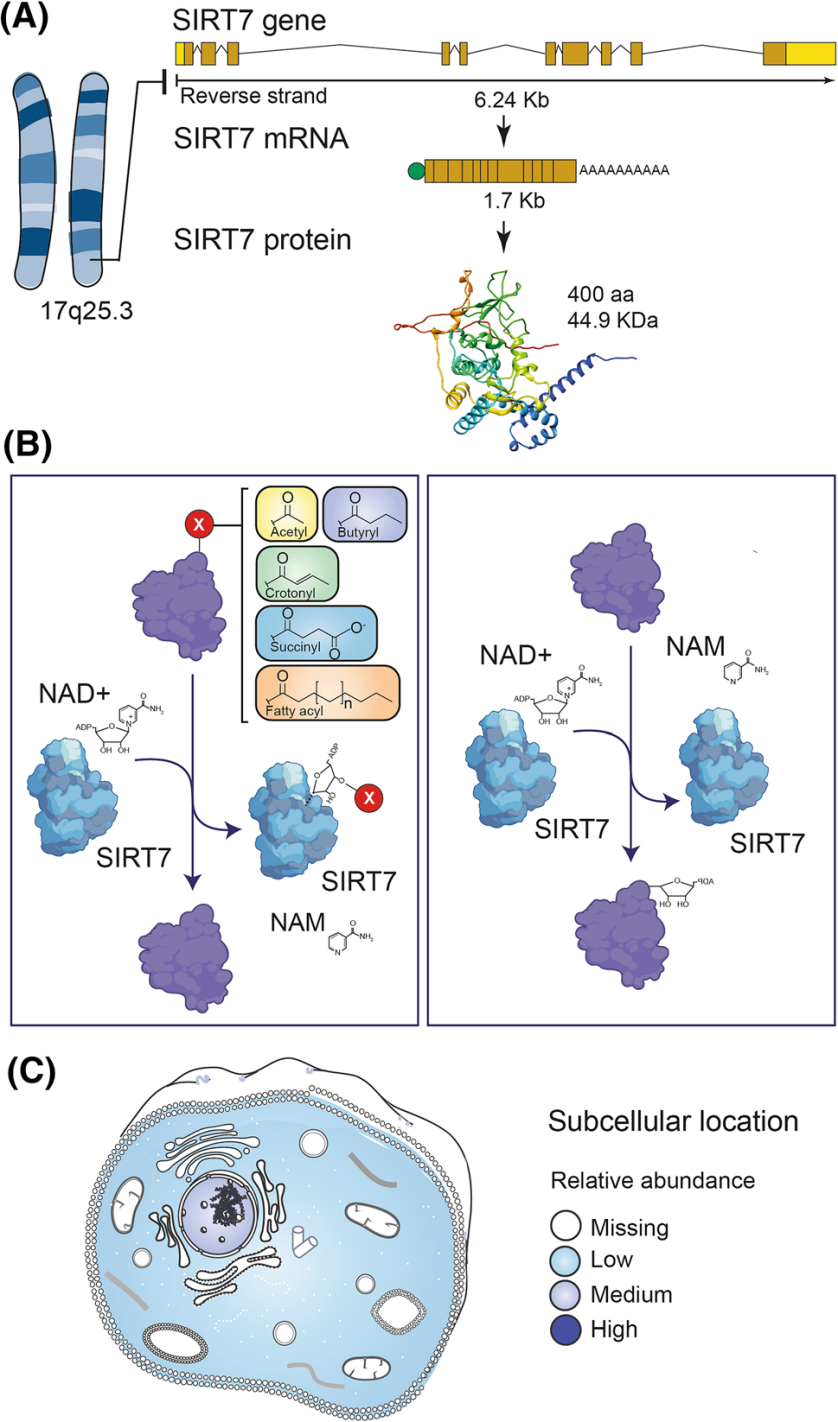
General characteristics of SIRT7. **A** The human SIRT7 gene is located on chromosomal region 17q25.3, spans a 6238 bp region of the reverse strand, and generates a 1725 bp transcript that encodes a 400 amino acid protein with a molecular weight of 44.9 KDa. **B** SIRT7 has been shown to carry out several enzymatic activities, all of which occur on lysine residues and require the presence of the co-substrate NAD+. NAD+ is consumed to generate nicotinamide (NAM). SIRT7 enzymatic activities are: (1) deacetylation, (2) debutyrylation, (3) decrotonylation, (4) desuccinylation, and (5) defatty-acylation that share a common mechanism of action where reaction intermediates are generated with the ADP-ribose group of NAD+ bound to SIRT7. Subsequent reactions regenerate SIRT7 and release the ADP-ribose and the functional group that was removed. (6) ADP-ribosylation in which NAD+ acts as a donor of the ADP-ribose moiety for target proteins. **C** SIRT7 can localize to the nucleus and is the only sirtuin highly enriched in nucleolar compartments. Only a small proportion of SIRT7 remains in the cytoplasm

The human SIRT7 gene is located on chromosomal region 17q25.3. It is a single copy gene that spans a 6238 bp region of the reverse strand and generates a 1725 bp transcript. This transcript has ten exons, ranging in size from 71 bp (exon 4) to 676 bp (exon 10), and is translated into a protein of 400 amino acids and a molecular weight of 44.9 KDa [11] (Fig. 1A).

SIRT7, like other sirtuins, has preserved the central catalytic region, while the flanking N- and C-terminal regions are unique [12]. The catalytic core region adopts an elongated shape containing a large and structurally homologous Rossmann-fold domain, characteristic of NAD+/NADH-binding proteins; a smaller zinc-binding domain; and several loops connecting the two domains. These loops form a sharp, extended cleft between the large and small domains where NAD+ and peptide substrates containing acetyllysine enter from opposite sides and bind to the enzyme [13]. It should be noted that within the SIRT7 catalytic domain, residues S111 and H187 are responsible for deacetylation activity [13–15]. Notably, SIRT7 and SIRT6, compared to other sirtuins, lose a large helix bundle at the NAD+-binding Rossmann fold that connects the catalytic domain to the zinc-binding domain. This modification has been suggested to significantly reduce the flexibility of the structure and cause low deacetylase activity. Likewise, a rearrangement occurs in the middle of the structure that creates a large pocket, located next to the primary catalytic site, which has been proposed to act as a secondary active site for other types of reactions [16]. In this regard, SIRT7 has been reported to exhibit deacetylase [14], mono-ADP-ribosylase [16], desuccinylase [17], debutyrylase [18], defatty-acylase [19], and decrotonylase [20] activities (Fig. 1B), and apparently these are enhanced by the presence of double-stranded nucleic acids (dsDNA or dsRNA), especially transfer RNAs (tRNAs) [19, 21]. On the other hand, the N- and C-terminal regions have been shown to be important for SIRT7 interaction with other proteins, RNA, and DNA [19, 21, 22]. Furthermore, due to the presence of nuclear localization sequences (NLS) and nucleolar localization sequences (NoLS) in the N- and C-terminal regions, SIRT7 can localize to the nucleus and is the only sirtuin highly enriched in nucleolar compartments. Only a small proportion of SIRT7 remains in the cytoplasm (Fig. 1C) [23, 24].

The high presence of SIRT7 in the nucleoli should be highlighted and also that its permanence in these depends mainly on the ongoing transcription and the nascent pre-ribosomal RNA (rRNA), presumably the rRNA stabilizes the association of SIRT7 with a subset of proteins, mainly those involved in translation and rRNA processing [25, 26]. SIRT7 has been reported to interact directly and indirectly with many other proteins, with the most abundant category being transcription mapped proteins (for both Pol I and Pol II), followed by chromatin remodeling proteins and proteins involved in the processes of ubiquitination and proteasomal degradation [15]. Interestingly, SIRT7 and SIRT6 share many of their interacting proteins, primarily those involved in DNA repair, chromatin assembly, and aging [27]. Regarding the functions of SIRT7, in general terms this protein regulates rRNA transcription [28, 29], participates in DNA repair and maintenance of genomic stability [4, 17, 30, 31], helps to cope with different stress conditions, such as energetic [32], metabolic [33] and oxidative stress, promotes proliferation [34–36], and in certain cells is involved in cell differentiation [37, 38]. Each of these will be discussed in more detail in the following sections.

## SIRT7 association with aging

**Fig. 2:**
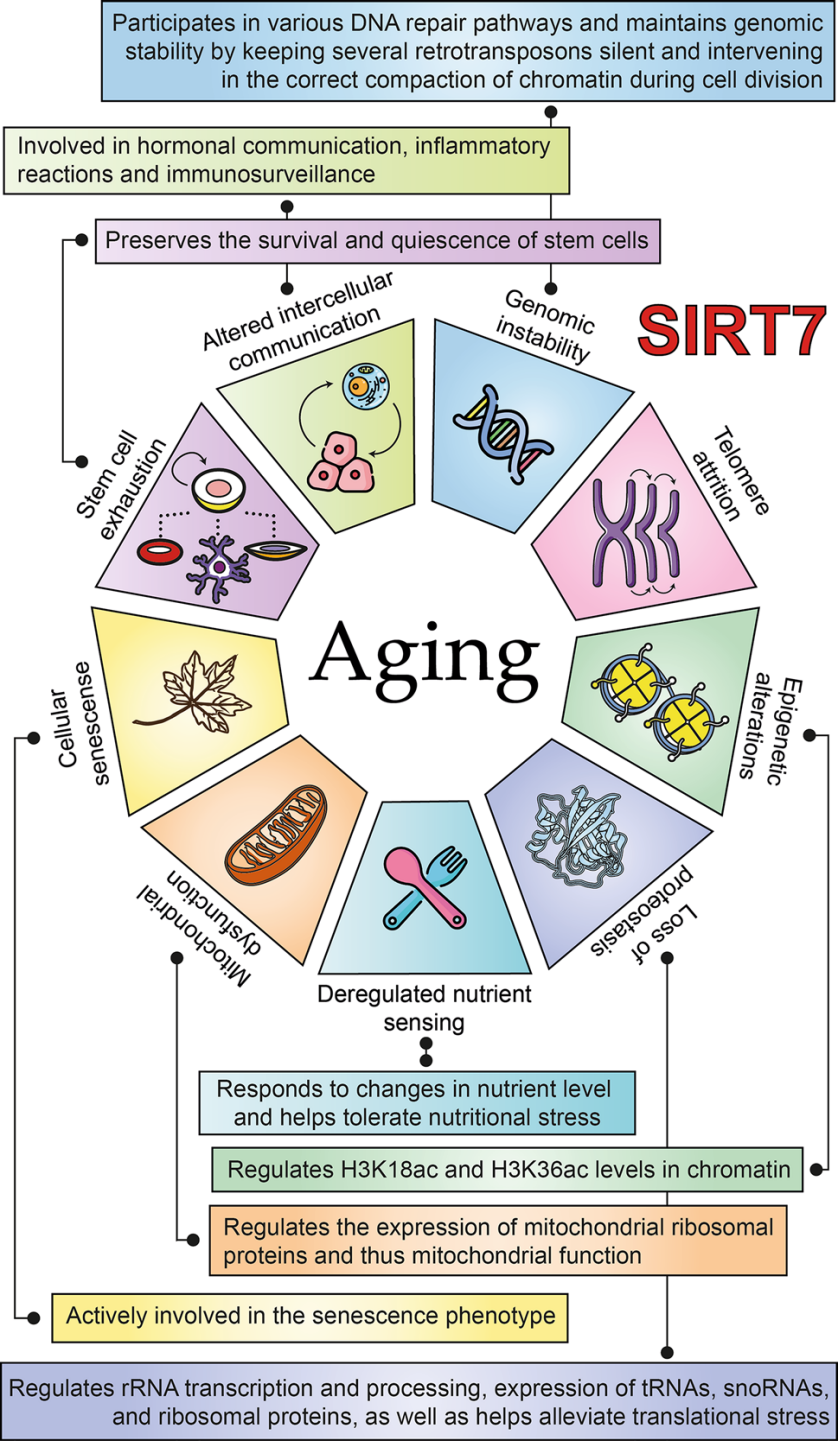
Association of SIRT7 with the hallmarks of aging

Consistent with the known roles of sirtuins in aging and life extension, several studies have established a close relationship between SIRT7 and age-related processes [34, 39] (Fig. 2). Some single nucleotide polymorphisms (SNPs) in the SIRT7 gene have been associated with longevity; for example, heterozygosity of SNP rs34829162 is a disadvantage for longevity compared to homozygotes of major and minor alleles [40]. Furthermore, significant reductions in SIRT7 levels have been reported in most organs and tissues of human and animal models as a consequence of aging, including the heart, liver, lungs, colon, skin, subcutaneous white adipose tissue (WAT) depots, hair follicles, and blood [41–46]. However, there are some tissues in which SIRT7 levels increase with aging, such as in the frontal lobe of the brain and retroperitoneal WAT depots [43, 47]. This could indicate that these changes are tissue specific, but it should be noted that the availability of NAD+ decreases with age [48], which, regardless of the levels in the different tissues, globally reduces the activity of SIRT7 and the rest of the enzymes that require this co-substrate. Meanwhile, SIRT7-deficient mice showed several signs of aging, including kyphosis, loss of subcutaneous fat, degenerative cardiac hypertrophy, severe osteopenia, and poor resistance to stress. All this led them to present a premature death around the year of age [41, 49]. Likewise, in a murine model of Hutchinson–Gilford progeria (a premature aging syndrome), an increase in the proteosomal degradation of SIRT7 was found due to a destabilization caused by progerin, a partially deleted form of nuclear lamin A. On the contrary, SIRT7 re-expression improved the phenotype and extends life span of these mice [50]. Although there are multiple ways to describe the molecular mechanisms that associate SIRT7 with aging and its associated diseases, in this review they were separated according to their participation in the hallmarks of aging described by López-Otín et al. [51].

### Link of SIRT7 with the hallmarks of aging

#### Genomic instability

**Fig. 3:**
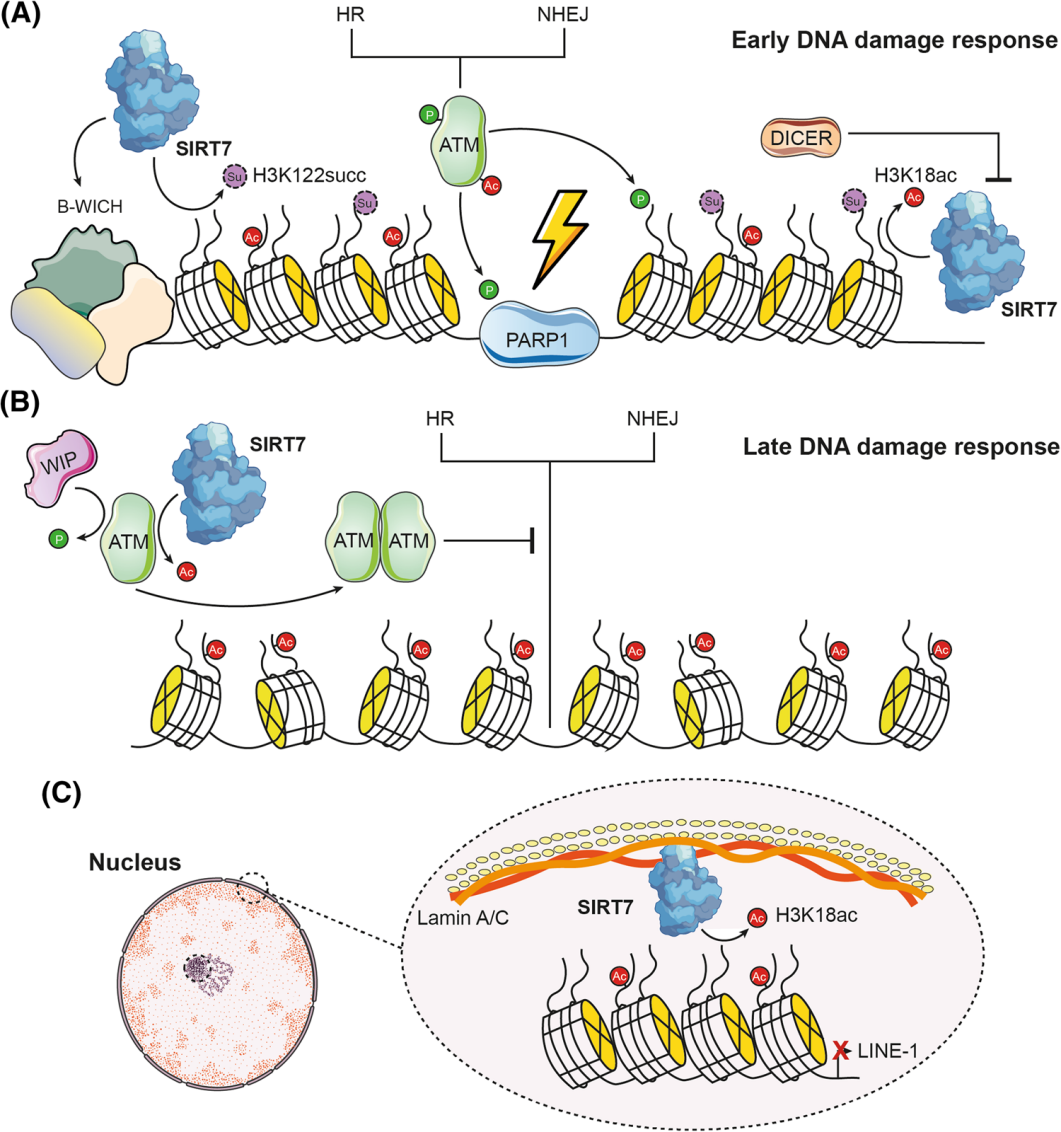
SIRT7 cooperates to maintain genomic stability. **A** SIRT7 is recruited to DNA damage sites in a PARP1-dependent manner and, in the early phase of the damage response, modulates H3K18ac and H3K122succ levels and interacts with the B-WICH complex to transiently compact chromatin and enhance DDR signaling. SIRT7 egress from damaged sites occurs in response to increased DICER levels, allowing chromatin to relax and components of the repair machinery (either homologous recombination or non-homologous end-joining machinery) to enter and perform their function. **B** In the final stage of repair, SIRT7 deacetylates ATM to promote its dephosphorylation by WIP1 and subsequent dimerization. In this way, the damage is resolved and the components of the repair machinery are removed. **C** SIRT7 also reduces the levels of H3K18ac in retrotransposons such as LINE-1 and promotes its association with lamin A/C in peripheral nuclear heterochromatin, thus preventing its expression and reducing the cumulative DNA damage it could generate

DNA repair systems are a complex network of mechanisms that together are capable of coping with most of the damage inflicted on our genome, whether by exogenous physical, chemical or biological agents, or by endogenous processes including DNA replication errors, spontaneous hydrolytic reactions and reactive oxygen species (ROS) [52]. A common denominator of aging is the accumulation of genetic damage and a considerable decrease in the efficiency of its repair. In addition, numerous diseases of premature aging, such as Werner's syndrome and Bloom's syndrome, result from an increased accumulation of DNA damage [53]. SIRT7 has been reported to be rapidly and transiently recruited in a PARP1-dependent manner to DNA damage sites and is required for efficient double-strand break (DSB) repair via homologous recombination (HR) and non-homologous end joining (NHEJ) [4, 17, 30, 31]. Indeed, SIRT7 deficiency in murine models was previously reported to cause replicative stress and NHEJ pathway malfunction culminating in overall genome instability [30]. In this sense, SIRT7 modulates H3K18ac levels and interacts with the B-WICH complex at DNA damage sites and thus promotes chromatin compaction and 53BP1 recruitment to DSB ends (Fig. 3A) [30, 54]. Transient chromatin compaction is required to enhance DDR signaling and 53BP1 prevents exonucleases from digesting free broken ends [55]. Likewise, it has been proposed that SIRT7 can desuccinylate H3K122succ and this is a step that activates upstream parts of the DDR and promotes chromatin compaction [17]. It is possible that SIRT7 egress from damage sites is in response to increased DICER levels [56]. In this way, the levels of H3K18ac increase at the site of damage and the chromatin relaxes so that the components of the repair machinery can carry out their function. Furthermore, SIRT7 is responsible for interacting with and deacetylating ATM, one of the main response factors to DSBs, in order to promote its dephosphorylation by WIP1 and consequent dimerization and deactivation in the final stages of DNA damage repair (Fig. 3B) [31]. This is very important as persistent ATM activation can induce senescence [57]. Meanwhile, in response to ultraviolet (UV) radiation damage, the catalytic activity of SIRT7 is enhanced by being phosphorylated by ATR and leading it to deacetylate NPM. Deacetylated NPM is excluded from the nucleolus and then binds to MDM2 to prevent ubiquitination and degradation of p53, one of the main guardians of the genome [58]. Possibly related to this, UV photoaging of human skin increases SIRT7 levels, while natural skin aging decreases them [46]. This could indicate persistent damage signaling, which leads to senescence. SIRT7's role in regulating rRNA expression is also of paramount importance in maintaining genomic stability because a high rate of rRNA transcription can create genomic instability. This is because ribosomal DNA (rDNA, in humans, located on the non-sex acrocentric chromosomes) is highly susceptible to losing genetic material (rDNA copies) due to its organization into long stretches of tandem repeats [29].

On the other hand, SIRT7 also helps prevent the expression of retrotransposons from the long interspersed nuclear element 1 (LINE-1) families [59, 60]. Recent evidence has suggested that LINE-1 activity contributes to a series of aging-related phenomena, mainly related to the generation of DNA damage during cutting and insertion processes, so its expression is strictly regulated. However, these mechanisms become less efficient during the aging process, resulting in LINE-1 derepression [61]. In this manner, SIRT7 reduces the levels of H3K18ac in these retrotransposons and promotes their association with lamin A/C in peripheral nuclear heterochromatin, thus preventing their expression and reducing the accumulated DNA damage that they could generate (Fig. 3C) [59]. In contrast, SIRT7 deficiency increases LINE-1 expression [60]. This same function is also carried out by SIRT6 [62].

Lastly, during cell division, SIRT7 deacetylates and activates HAT1, which in turn generates the high levels of H4K5ac and H4K12ac needed to recruit and ensure proper docking of CENP-A at centromeres. In accordance with this, SIRT7 deficiency is associated with an increase in aneuploidies [44, 63]. Furthermore, SIRT7 deacetylates DDX21 and this increases its helicase activity to prevent R-loop accumulation, thus safeguarding genome integrity [64]

#### Loss of proteostasis

SIRT7 levels are known to correlate positively with ribosome biogenesis, cell proliferation and viability, with its expression being abundant in metabolically active cells and low or even absent in non-proliferating cells [34–36]. Numerous studies have presented a direct connection between dysregulated ribosome biogenesis and aging, as well as small nucleoli are a cellular hallmark of longevity [28, 65]. In fact, it has been seen that an increase in ribosome biogenesis accelerates aging due to several factors, such as genomic instability created by the high rate of rRNA transcription, increased energy expenditure as a consequence of high protein synthesis and disruption of global proteostasis [28, 29].

**Fig. 4:**
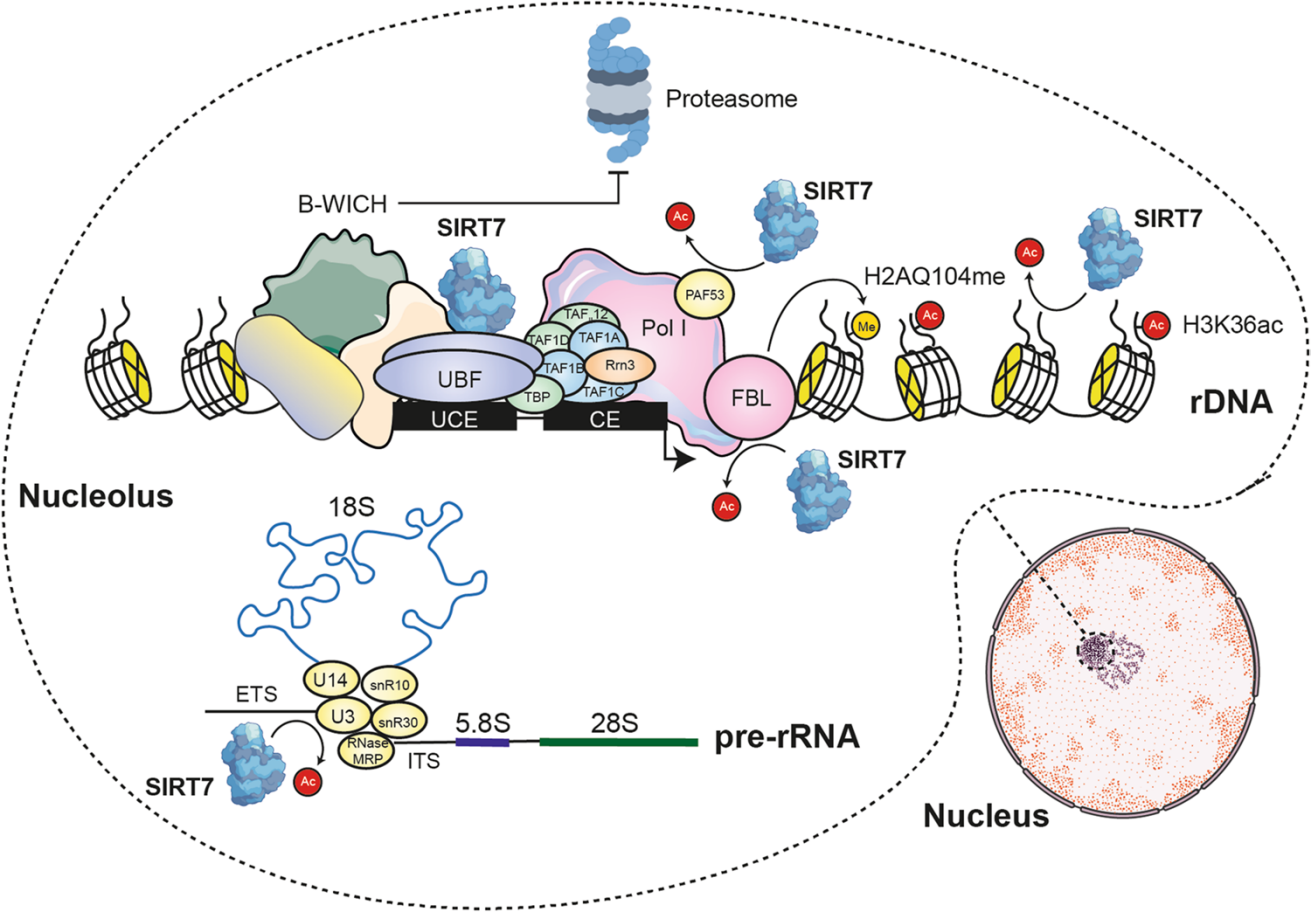
SIRT7 regulates rRNA levels. SIRT7 promotes the association of Pol I with rDNA by associating with the transcription factor UBF and deacetylating PAF53. Fibrillarin deacetylation by SIRT7 is also required to promote methylation of histone H2A (H2AQ104me), allowing chromatin to remain decondensed. On the other hand, SIRT7 helps in the cleavage of pre-rRNA by associating with the U3 snoRNP complex and deacetylation of its U3-55k subunit. To maintain a balance in rRNA levels, SIRT7 is responsible for deacetylation of H3K36ac at rDNA sequences and for anchoring the WICH complex at rDNA promoters, contributing to heterochromatin silencing and stability. B-WICH protects RPA194, the largest subunit of Pol I, from degradation

SIRT7 was initially thought to act only as a positive regulator of rRNA transcription, mainly involved in its biogenesis, but later it was discovered that it can also act as a negative regulator and is involved in multiple pathways that regulate pre-rRNA maturation and protein translation [22, 66–68] (Fig. 4). Initiation of rRNA transcription requires SIRT7 to associate with the transcription factor UBF and also deacetylate residue K373 of PAF53 (previously acetylated by CBP) to promote association of Pol I with rDNA [22, 25]. During interphase, SIRT7 is detected in the nucleoli, colocalizing with Pol I and transcriptionally active copies of rDNA, particularly in promoter regions [35]. It has been shown that SIRT7-dependent deacetylation of fibrillarin is also required during interphase for rRNA transcription because fibrillarin is thus activated and methylates histone H2A at residue Q104 (H2AQ104me), allowing chromatin remains decondensed [69]. At the onset of mitosis, nucleolar disassembly causes topological separation of SIRT7 and fibrillarin. SIRT7 is inactivated by being phosphorylated by CDK1-cyclin B and remains in the nucleolar organizing region (NOR) interacting with UBF. Meanwhile, fibrillarin is hyperacetylated by CBP, compromising H2AQ104 methylation and causing chromatin condensation and repression of rDNA transcription. SIRT7 reactivation requires its dephosphorylation to resume interaction with fibrillarin during telophase and, in turn, rDNA transcription [22, 69]. It should also be mentioned that SIRT7 deficiency causes a decrease in Pol I levels by decreasing the levels of RPA194, its largest subunit. Apparently, the B-WICH chromatin remodeling complex dissociates from the Pol I complex because its MYBBP1a, WSTF, and SNF2h subunits do not interact with SIRT7 and this facilitates Pol I degradation [15]. On the other hand, SIRT7 also plays an essential role in specific pre-rRNA cleavage because it associates with the U3 small nucleolar ribonucleoprotein (snoRNP) complex and deacetylates the U3-55k (Rrp9) subunit, thus promoting pre-rRNA cleavage at the 5′-terminal processing site, which represents the initial step for the processing of 18S rRNA [66]. Contrary to its role as an activator of rRNA transcription, SIRT7 is responsible for the deacetylation of H3K36ac in rDNA sequences, which contributes to the silencing and stability of rDNA heterochromatin [68]. As previously mentioned, SIRT7 interacts with the WICH complex, and in this regard, SIRT7 functions as a scaffold for the SNF2H subunit and stabilizes its binding to rDNA promoter sequences to promote heterochromatin formation and rRNA silencing, while the MYBBP1 subunit regulates this activity [54, 70]. Also, SIRT7 deacetylates H3K18ac and mediates transcriptional repression of a selected set of genetic targets such as RPS20, RPS7, RPS14, NME1, and COPS2, which are involved in RNA processing, protein translation, and cellular macromolecular metabolism [14]. All together would indicate that SIRT7 is one of the main proteins that maintains rRNA levels in balance, allowing the continuity of the cell cycle and proliferation but at the same time regulating energy expenditure and chromatin stability. For this reason, the decreased expression or activity of SIRT7 during aging could lead to a global imbalance in proteostasis.

SIRT7 is also known to play a role in protein synthesis because it has been identified associated with monoribosomes and its removal reduces protein synthesis rates more than rRNA synthesis rates [15, 67]. SIRT7 interacts with mTOR and the TFIIIC2 complex and, in this way, can target genetic regions transcribed by Pol III and trigger a reduction in tRNA levels, thus limiting translation [67]. In addition, SIRT7 also promotes Pol II transcription of small nucleolar RNAs (snoRNAs) and ribosomal protein messenger RNAs (mRNAs) by deacetylation and activation of CDK9, leading to release of P-TEFb from the 7SK small nuclear ribonucleoprotein (snRNP) complex and subsequent phosphorylation of the C-terminal domain of Pol II [26]. Taken together, we can say that SIRT7 is also important for protein translation and the decrease in its activity could be related, at least partially, to the global reduction in protein synthesis observed in aging [71].

#### Deregulated nutrient sensing

**Fig. 5:**
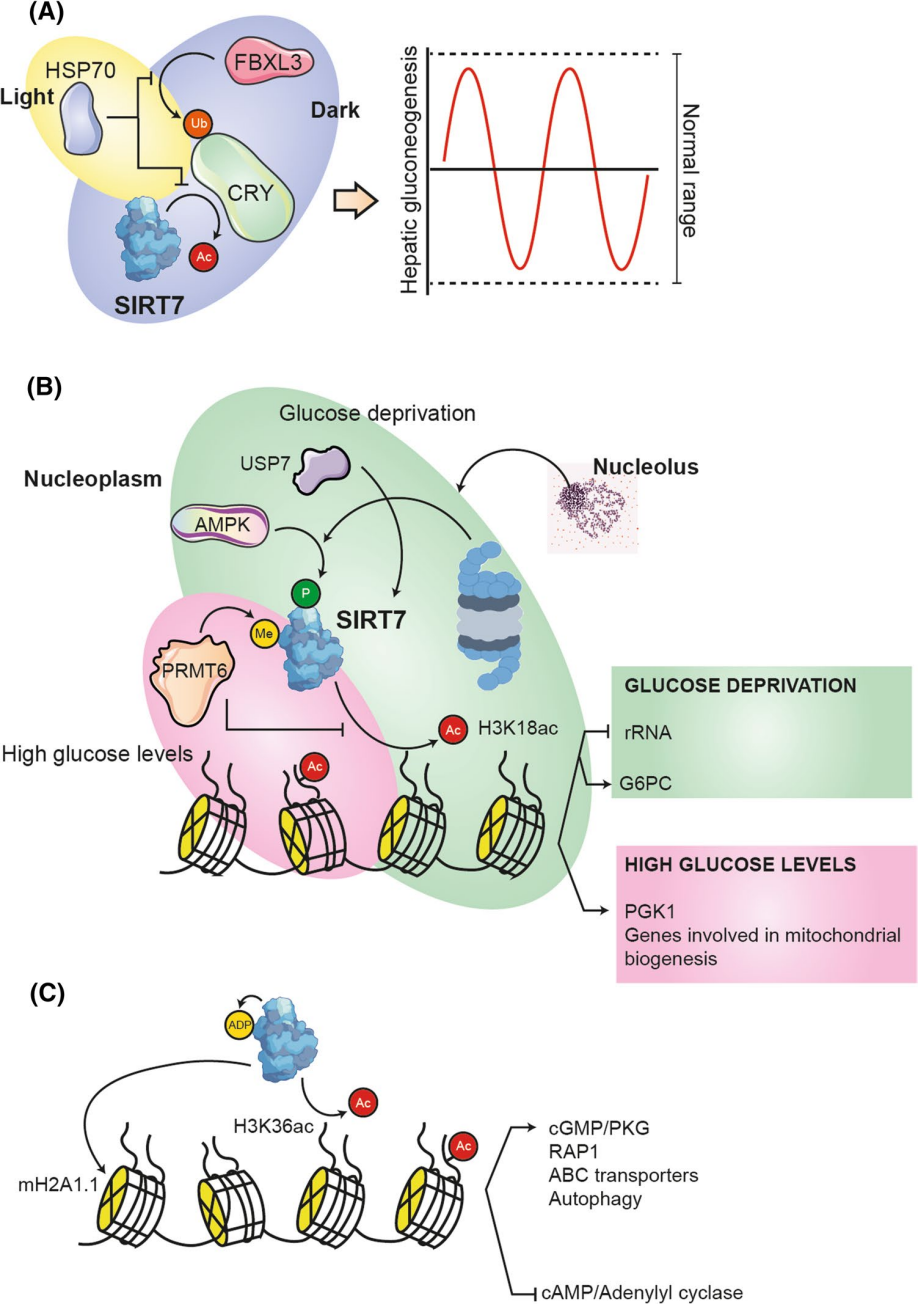
Participation of SIRT7 in the detection of glucose availability. **A** Liver SIRT7 is involved in the regulation of circadian glucose homeostasis and rhythmic hepatic gluconeogenesis. During the dark phase, SIRT7 deacetylates CRY1 and promotes its ubiquitination by FBXL3 and subsequent degradation. During the light phase, HSP70 levels increase and interact with SIRT7 to promote its ubiquitination and degradation. **B** When glucose levels are high, PRMT6 methylates SIRT7 to reduce its deacetylase activity and increase H3K18ac in PGK1 and mitochondrial biogenesis. During glucose deprivation, REGγ regulates the redistribution of SIRT7 from the nucleolus to the nucleoplasm and also promotes degradation in an AMPK phosphorylation-dependent manner. In this way, rRNA transcription is reduced and H3K18ac is increased in G6PC. **C** During metabolic stress, SIRT7 self-monoADP-ribosylates to interact with mH2A1.1. and modify the expression of a subset of genes, mainly second messengers

Anabolic signaling has been reported to accelerate aging, while decreased nutrient signaling prolongs longevity [72]. Since the activity of sirtuins depends on the presence of NAD+, they can detect when nutrient and energy levels are low (coinciding with an increase in NAD+) or high (coinciding with a decrease in NAD+) [73]. In the particular case of SIRT7, its participation in sensing glucose availability has been reported. Liver SIRT7 is involved in the regulation of circadian glucose homeostasis and rhythmic hepatic gluconeogenesis. To achieve this, during the dark phase, SIRT7 deacetylates residues K565 and K579 of CRY1, a central clock protein, and promotes their ubiquitination by FBXL3 and subsequent degradation. Meanwhile, increasing body temperature as an early response to light-driven timing signals induces transcription of HSP70, which interacts with SIRT7 to promote its ubiquitination and proteasomal degradation, while simultaneously protecting CRY1 (Fig. 5A) [33]. SIRT7 also negatively regulates the activity of PGK1, an important enzyme in the metabolic glycolysis pathway, by deacetylating its K323 residue [74]. When glucose levels are high, PRMT6 methylates SIRT7 at residue R388 to reduce its deacetylase activity and increase H3K18ac at the promoters of its target genes, such as those involved in mitochondrial biogenesis (Fig. 5B). This mechanism helps maintain energy homeostasis [75]. Furthermore, the interaction of SIRT7 and its negative regulator USP7 regulates gluconeogenesis by modulating G6PC expression according to the glucose levels [76]. To maintain glucose homeostasis during metabolic stress, SIRT7 self-monoADP-ribosylates (where residue N189 is key in this reaction), and this post-translational modification allows it to interact with mH2A1.1. (a histone variant involved in transcriptional repression) and is recruited in intergenic regions of a subset of genes, many of them involved in second messenger signaling, resulting in their specific up- or downregulation (Fig. 5C) [16]. Likewise, SIRT7 redistributes from nucleolus to nucleoplasm after glucose deprivation, reducing rDNA transcription and energy consumption [25]. REGγ has been reported to regulate this redistribution and also promotes SIRT7 degradation in an AMPK phosphorylation-dependent manner (Fig. 5B) [77]. For all these reasons, deficiencies in SIRT7 due to aging could play a role, to a still unknown degree, in the age-dependent reduction of glucose tolerance and carbohydrate metabolism.

Findings have also been found on the role of SIRT7 in the regulation of lipid metabolism, particularly in the liver, but some of them are contradictory. On one side, SIRT7 deficiency was reported to prevent hepatic steatosis by a high-fat diet. For this, it was described that SIRT7 interacts with the DCAF1/DDB1/CUL4B E3 ubiquitin ligase complex and prevents the degradation of TR4, which leads to the expression of key genes for the uptake and synthesis of fatty acids such as CD36, MOGAT1, CIDEA and CIDEC [78]. SIRT7 can also desuccinylate residue K287 of PRMT5 to prevent its interaction with the E3 ubiquitin-protein ligase STUB1 and, instead, favor its binding to MEP50 and increase its methyltransferase activity. In this way, the PRMT5-MEP50 octamer methylates the SREBP1e transcription factor and increases the levels of cholesterol, fatty acids and triglyceride biogenesis in cells [79]. Another study reported that SIRT7 deficiency produced hepatic microvesicular steatosis, with elevated plasma triglycerides and free fatty acids, and linked it to impaired mitochondrial function [80]. On the other hand, contradicting the above, SIRT7 was described as preventing the development of fatty liver and steatosis after an unfolded protein response in the endoplasmic reticulum (UPR^ER^) such as those caused by hypercaloric diets. The mechanism pointed out here was through MYC recruiting SIRT7 to ribosomal protein promoters and suppressing its expression, thereby relieving endoplasmic reticulum stress [81]. Due to all these controversies, further studies are needed to reach a conclusion on the role of SIRT7 in hepatic lipid metabolism.

#### Mitochondrial dysfunction

**Fig. 6:**
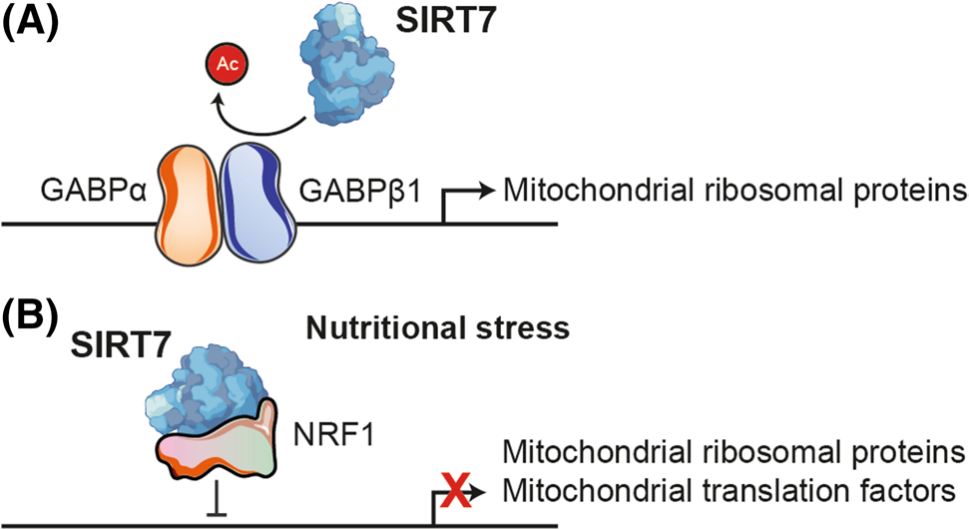
Role of SIRT7 in mitochondrial function. **A** SIRT7 promotes the transcription of nuclear-encoded mitochondrial genes by deacetylating GABPβ1 and facilitating the formation of the GABPα/GABPβ heterotetramer. **B** Under conditions of nutritional stress, SIRT7 specifically binds and suppresses NRF1 activity at the promoters of mitochondrial ribosomal proteins and mitochondrial translation factors

Mitochondrial dysfunction, including decreased oxidative capacity and increased oxidative damage, is thought to contribute substantially to biological aging [32]. SIRT7 can improve mitochondrial function and its deficiency causes multisystem mitochondrial dysfunction and disrupts oxidative phosphorylation. This is explained because SIRT7-mediated deacetylation of GABPβ1 residues K69, K340, and K369 facilitates formation of the GABPα/GABPβ heterotetramer and transcriptional activation in nuclear-encoded mitochondrial genes, including promoters of mitochondrial ribosomal proteins (Fig. 6A) [80]. Mitochondrial ribosomes synthesize 13 key subunits of the oxidative phosphorylation system. In this regard, the lack of SIRT7 inevitably impairs the oxidation process, decreases cellular energy production, and increases ROS production in a similar way to natural aging [82]. Furthermore, as mentioned above, this function of SIRT7 regulating mitochondrial ribosomal protein transcription is important in the modulation of glucose levels, in lipid metabolism and in the maintenance of energy homeostasis [75, 81]. Likewise, under conditions of nutritional stress, SIRT7 specifically binds and suppresses NRF1 activity at the promoters of mitochondrial ribosomal proteins and mitochondrial translation factors to cause transcriptional repression of these genes (Fig. 6B). This relieves mitochondrial protein folding stress (PFS^mt^) and reduces cell proliferation, an extremely important mechanism for stem cell survival and maintenance [83, 84], which will be discussed later in this review.

#### Cellular senescence

Cellular senescence can be defined as stable cell cycle arrest coupled with stereotyped phenotypic changes, such as metabolic reprogramming, chromatin rearrangement, high levels of β-galactosidase, and the persistent presence of DNA damage markers [85, 86]. Basically, all of the processes mentioned here (genomic instability, telomere attrition, loss of proteostasis, dysregulated nutrient sensing, and mitochondrial dysfunction), together or separately, can lead to cellular senescence [51]. In this way, SIRT7 may be related to senescence in different ways and mechanisms. For example, SIRT7 levels in the nucleolus decrease with senescence [23], while overexpression can mitigate senescence [87]. Different stress conditions (osmotic, thermal, nutritional, transcriptional and translational) have been shown to cause the release of SIRT7 from the nucleolus to the nucleoplasm with the consequent reduction in the levels of pre-rRNA and its processing products [25, 66]. The integrated stress response (ISR) is responsible for this to reduce energy expenditure and give the cell time to face adverse conditions and restore homeostasis that allows it to survive [88]. Notably, ISR activity increases with age and it has been seen that if it is maintained for long periods of time, it limits life expectancy despite reducing ribosomal biogenesis, since it leads to cellular senescence [89]. Oxidative damage, inflammation and high glucose levels caused senescence in endothelial cells, which was associated with a decrease in SIRT7 levels and as a result an increase in miR-335-5p levels [90]. In this regard, SIRT7, like other sirtuins, is susceptible to the effects of endogenous ROS, particularly hydrogen peroxide, which cause its carbonylation and subsequent autophagic degradation [91]. On the other hand, with the number of passages, the cultures of primary mammary epithelial cells increased the expression of SIRT7, as well as the number of cells positive for β-galactosidase and with high levels of expression of p16^INK4a^ and p21^CIP1^ [92]. The same thing happened with senescent fibroblasts, where it was also explained that this occurs, in part, because SIRT7 descrotonylates the K25 residue of PHF5A and causes an abnormal alternative splicing of CDK2, where an intron is retained, and therefore lowers its levels [20]. Another aspect to consider here is that excessive cell proliferation triggers rDNA instability, with cleavage and loss of rDNA gene copies, which in turn induced a senescent phenotype that is independent of telomere shortening [54]. Although not proven, in humans, given the genomic proximity of rRNA copies and telomeres, SIRT7 could also play a role in maintaining heterochromatin in the latter and providing stability, at least in non-sex acrocentric chromosomes (chromosomes 13, 14, 15, 21 and 22) [68].

#### Stem cell exhaustion

Stem cells are cells with the ability to self-renew and also differentiate, so they are important for replacing damaged cells and maintaining tissue populations [93]. Stem cell exhaustion develops as the integrative consequence of multiple types of damage associated with aging and is probably one of the main culprits in the aging of tissues and the body by limiting their ability to regenerate [51]. SIRT7-deficient mice showed an increase in hematopoietic stem cells (HSCs) in the bone marrow, but these cells had a reduced ability to repopulate the lymphoid compartment of lethally irradiated mice compared to wild-type bone marrow cells [30]. This is because SIRT7 inhibition of the transcription of mitochondrial ribosome components is relevant in preventing HSCs from losing their quiescence and consequently proliferating and reducing their survival [83, 84]. Likewise, SIRT7 maintains telogen quiescence (resting) in hair follicle stem cells and prevents them from entering the anagen phase (growth). This is explained by the fact that SIRT7 deacetylates residue K612 of NFATc1 and favors its phosphorylation by GSK-3β with the consequent inhibition of its signaling and its subsequent nuclear degradation via PA28c [45]. SIRT7 deficiency in human mesenchymal stem cells (hMSCs) contributes to the appearance of several senescence-related features such as accelerated functional wasting due to decondensation of heterochromatin in the nuclear periphery and activation of the cGAS-STING pathway and retrotransposons such as LINE1 [60]. Meanwhile, SIRT7 is important for intestinal epithelial homeostasis and its role is especially important in maintaining LGR5+ stem cells for epithelial regeneration. Its deficiency leads to dysregulated chromosome segregation, a poor response to DNA damage and inadequate differentiation of intestinal cells, while increasing WNT signaling and the expression of genes associated with colorectal cancer [44]. In human dental pulp stem cells (HDPSCs), it has been described that miR-152 increases with cell aging and promotes the senescent phenotype through its regulatory role on SIRT7 levels [94]. SIRT7 deletion promotes osteogenic differentiation of bone marrow mesenchymal stem cells through activation of the WNT/β-catenin signaling pathway and by increasing H3K18ac levels at the OSX transcription factor promoter and thus promote its expression, particularly of isoforms 1 and 2 [95, 96]. SIRT7 binding to the OSX promoter is guided by RBM6 and regulated by long non-coding RNA (lncRNA) PLXDC2-OT [96]. Low levels of SIRT7 were also found in induced pluripotent stem cells (iPSCs) with prolonged in vitro culture, which recapitulate several aspects reported for premature aging syndromes (for example, Hutchinson-Gilford progeria syndrome) and for somatic cell senescence [97]. Although this action of SIRT7 to preserve stem cell quiescence is very important to maintain their population, it can cause problems in processes where stem cells are required to differentiate and therefore proper regulation is necessary. For example, downregulation of SIRT7 by exosomes secreted by bone marrow mesenchymal stem cells carrying miR-125b enhances the response to myocardial ischemia–reperfusion injury [98], possibly by allowing damaged cells to be replaced more rapidly and properly. Similarly, exosomal miR-17-5p derived from human umbilical cord mesenchymal stem cells improves ovarian function in premature ovarian failure by downregulating SIRT7 [99]. SIRT7 overexpression in mouse embryonic fibroblasts inhibited cell growth and proliferation by activating p53- and c-MYC-dependent transcription [100].

#### Altered intercellular communication

Aging implies changes in intercellular communication, be it endocrine, neuroendocrine or neuronal. Overall, neurohormonal signaling tends to be deregulated, inflammatory and immunosurveillance reactions against pathogens and premalignant cells decrease, and the composition of the pericellular and extracellular environment changes [51]. Related to the fibrotic remodeling that the aging heart undergoes, cardiac fibroblasts after stimulation with angiotensin-II (Ang-II) increase the expression and phosphorylation of SIRT7 and this causes an increase in their proliferation, differentiation into myofibroblasts, and extracellular matrix (ECM) deposition [101]. Furthermore, SIRT7 antagonizes TGF-β signaling by deacetylating residue K428 of SMAD4 and causing it to be ejected from the nucleus and subsequently degraded by β-TrCP1. As feedback, TGF-β signaling antagonizes SIRT7 expression by causing SMAD3/4 to recruit HDAC8 to the SIRT7 promoter and thus chromatin to be remodeled through H4 deacetylation [102, 103]. Meanwhile, SIRT7 expression in granulosa-lutein cells from women without ovarian factor was higher with increasing age and was significantly higher in women who responded poorly to in vitro fertilization (IVF) [104]. Regarding the immune system, SIRT7 silencing reduced lipopolysaccharide (LPS)-induced pro-inflammatory effects and induced an endomesenchymal transition that increased endothelial barrier permeability. All this is a consequence of the suppression of NFκB signaling and the resulting low levels of ICAM1, VCAM1, IL8, IL6, cadherin, and PECAM1, but increases in collagen, αSMA, TGFβR1, and SNAIL [105]. SIRT7 interacts with RAN and deacetylates it at residue K37, leading to decreased binding between RAN and nuclear export components and, as a consequence, retention of NF-κB p65 in the nucleus [106]. SIRT7 is also involved in exosomal circ-RPS5-mediated microglial M2 (immunoregulatory) switching under LPS conditions [107]. In response to IFNγ, SIRT7-deficient hepatocellular carcinoma cells increased MEF2D acetylation levels and consequently PD-L1 levels. High PD-L1 expression caused T-cell exhaustion and also reduced cancer immunosurveillance [108]. All this indicates that SIRT7 tends to promote inflammatory processes, which are pronounced during aging.

## Aging-associated diseases

### Cardiovascular diseases

Age plays a vital role in the deterioration of cardiovascular (CV) functionality, resulting in an increased risk of cardiovascular diseases (CVD) such as atherosclerosis, stroke and myocardial infarction [109]. SIRT7-deficient mice exhibit a marked pathologic cardiac remodeling with an increase in cardiac fibrosis, enlarged cardiomyocytes with marked variations in caliber (indicating ongoing cardiac remodeling), inflammatory infiltrates in the myocardium, accumulation of lipofuscin, and increased number of apoptotic cells (induced by the intrinsic and extrinsic pathway), increased number of granulocytes, T cells and elevated levels of IL-12 and IL-13 in the blood [41, 110]. One study reported that the marked presence of cardiac hypertrophy and fibrosis in these mice is due to the loss of SIRT7 interaction with GATA4. Thus, GATA4 is hyperacetylated and activated, causing an increase in the expression of its target genes, such as the natriuretic factors ANF and ANFB [110]. Elderly patients with cardiovascular disease and deficiency of plasma selenium, a trace mineral that enhances antioxidant capacity and influences inflammatory signaling pathways, showed low levels of SIRT7 in peripheral blood mononuclear cells (PBMCs) [111]. In human aortic smooth muscle cells, curcumin caused a decrease in the expression of SIRT7 and consequently there was a decrease in the number of nucleoli and an increase in their size that led to a decrease in cell proliferation [112]. Patients with atherosclerosis were found to have low blood levels of p53, lincRNA-p21, and SIRT7, but elevated levels of miR-17-5p. Based on this, p53-dependent expression of lincRNA-p21 was reported to downregulate miR-17-5p in vascular smooth muscle cells (VSMCs), thereby upregulating SIRT7 which, in turn, enhances activation of the WNT/β-catenin pathway and protects against atherosclerosis progression [113, 114]. Similarly, the lncRNA SNHG7-003 can inhibit VSMC proliferation, migration, and invasion after oxidized low-density lipoprotein (ox-LDL) treatment by capturing miR-1306-5p and thus protecting SIRT7 mRNA [115]. Lastly, compared with healthy controls, lung fibroblasts from patients with idiopathic pulmonary fibrosis, as well as from patients with systemic sclerosis-associated interstitial lung disease, showed lower levels of SIRT7, and the same was observed in the bleomycin mouse model of pulmonary fibrosis. SIRT7 appears to downregulate type I collagen and alpha-smooth muscle actin (α-SMA) by attenuating TGF-β signaling through reduction in SMAD3 levels [42]. With all this we can mention that SIRT7 is key in the predisposition to CVDs in aging.

### Obesity

Obesity-related diseases appear to be associated with the acceleration of cellular processes seen during normal aging [116]. SIRT7 levels were higher in adipose tissue from obese patients than in normal weight individuals [117]. In this regard, SIRT7 has been shown to act as an important driver of adipogenesis and adipogenic differentiation, since its deficiency in adipogenic cells prevents the correct formation of WAT. For this, SIRT7 is regulated by miR-93 and SIRT7, in turn, regulates SIRT1 by binding to it and preventing its self-deacetylation of the K230 residue, possibly through steric hindrance, which causes a partial inhibition of its catalytic activity. This inhibition of SIRT1 activity is important in keeping H4K16ac and H1K26ac in the PPARγ promoter and allowing their transcription [37, 38]. Furthermore, SIRT7 binds to and deacetylates PPARγ2 at residue K382, which is important in adipocytes as this step promotes the rosiglitazone-dependent transcriptional activity of PPARγ2, leading to expression of a suite of genes involved in lipogenesis, including SREBP1C, ACACA, FASN and SCD1 [118]. Furthermore, brown adipose tissue (BAT) was also decreased in SIRT7-deficient mice and their body temperature increased in association with elevated levels of UCP1 and DIO2, genes involved in thermogenesis [78]. There are many other mechanisms in which SIRT7 could indirectly be linked to obesity, such as lipid and glucose metabolism, mitochondrial and endoplasmic reticulum stress, which were mentioned above.

### Cancer

Cancer and aging can be considered as two different manifestations of the same underlying process (accumulation of cell damage) and, therefore, both are closely related [51, 119, 120]. SIRT7 is overexpressed in a wide variety of cancers such as hepatocellular carcinoma [121], gastric cancer [122], breast cancer [92], ovarian cancer [123], bladder cancer [124], colorectal cancer [125], and glioblastoma multiforme [126], among many others. This is possibly because it helps cancer cells proliferate and deal with DNA damage and various other forms of stress to which they may be subjected (e.g., chemotherapy, radiation, metabolic stress, hypoxia).

SIRT7 expression was significantly higher in breast cancer tumor compared to adjacent normal breast tissue and was also correlated with higher histologic grade and poor prognosis [92, 127]. Overexpression of miR-3666, whose target is SIRT7, reduced the proliferation, promoted apoptosis, and increased drug sensitivity by activating p38^MAPK^ [128, 129]. Regarding immunosurveillance, SIRT7 has also been found to be associated with infiltration of M1 macrophages (inflammatory) and exhausted T cells in luminal breast cancer [130]. Apparently, SIRT7 favors the development of breast cancer but not the development of metastasis. Indeed, SIRT7 mRNA levels gradually decreased in patients as they progressed to more advanced metastatic stages [131]. SIRT7 dysregulation was associated with breast cancer lung metastases due to overactivation of TGF-β signaling that promotes epithelial-to-mesenchymal transition [103]. Despite the oncogenic effects of SIRT7, the high levels of this protein in breast cancer cells make them susceptible to the effects of fasting, where SIRT7 is phosphorylated at residue T263 by AMPK and then at residue S259 by GSK3β to prevent its binding to E3 ligase UBR5 and subsequent polyubiquitination and degradation. Hyperphosphorylated SIRT7 can bind to and stabilize SKP2, preventing its EGF-induced protein turnover and subsequently compromising AKT activation [132].

In hepatocellular carcinoma, SIRT7 is overexpressed due to transcriptional repression of miR-125a-5p, miR-125b and miR-526b through promoter methylation and also due to overexpression of circPVT1 which sponges miR-3666 [121, 133, 134]. This overexpression stimulates proliferation and cell cycle progression by promoting rRNA and cyclin D1 transcription, while inhibiting p21^WAF1/CIP1^ transcription [121]. SIRT7 also interacts with p53 to decrease its binding to the NOXA promoter and reduce its transcription, thereby inhibiting doxorubicin-induced apoptosis [135]. Likewise, SIRT7 reduces H3K18ac levels at the organic anion transporter OAT2 promoter and downregulates its expression, thus reducing 5-fluorouracil (5-FU) uptake [136]. In contrast, 5-FU promotes SIRT7 degradation by activating the TBP1 proteasome-dependent pathway and promoting its degradation [137].

SIRT7 levels are increased in lung cancer tumor tissues and cell lines, and these levels were also associated with clinicopathologic features, including positive lymph node metastases, high pathologic stage, and large tumor size. SIRT7 was found to activate AKT and ERK1/2 signaling pathways, upregulate cyclin D1, cyclin E1, CDK2, and CDK4, and downregulate p21 and p27. It also favored the EMT transition by increasing the levels of N-cadherin, vimentin, SNAIL, and SLUG and reducing those of E-cadherin [138]. Increased expression of miR-125a-5p and/or miR-3666 was found to cause decreased cell growth and increased radiosensitivity in non-small cell lung cancer cells by reducing SIRT7 levels [139, 140]. Similarly, SIRT7 depletion sensitizes these cells to gemcitabine therapy by inhibiting autophagy [141].

SIRT7 was overexpressed in glioblastoma multiforme tumors compared to matched adjacent non-tumor tissues and mainly in those of high grade. The same thing happened in glioma cell lines where it was also found that SIRT7 promotes proliferation and improves colony formation and invasion capacity, this linked to the activation of the STAT3 and ERK signaling pathways and the overexpression of MMP9 and CDK2 [126]. In addition, in this cancer TDO2 helps to recruit SIRT7 in the chromatin after DNA damage by chemotherapeutic agents, which transiently decreases histone H3K18 acetylation and increases damage signaling and repair [142].

In colorectal cancer patients, SIRT7 level was significantly correlated with tumor stage, lymph node metastasis, and poor patient survival. SIRT7 promotes cancer cell proliferation, colony formation, and motility by activating MAPK, ERK, and MEK pathways, increasing vimentin and fibronectin expression, and reducing E-cadherin and β-catenin expression [125].

In melanoma, the expression of miR-148b is downregulated, while that of SIRT7 is upregulated, favoring its proliferation, migration, and invasion [143]. Meanwhile, in the SK-MEL-28 and A375 cell lines, in vitro and in BALB/c mouse xenografts, SIRT7 was found to participate in the prevention of DNA damage and is regulated by circZNF609 through the sponge miR-138-5 p [144].

### Bone diseases

Bone diseases are a common problem in the elderly due to changes in bone and cartilage architecture, porosity, and composition [145]. SIRT7 deficiency leads to the development of severe osteopenia due to inadequate osteoblastic differentiation. This is the result of decreased OSX transcription factor activity due to loss of interaction with SIRT7. Under normal conditions, SIRT7 deacetylates residue K368 at the C-terminus of OSX, allowing SIRT1 to depropionylate its N-terminus, an important step for its transactivation [49]. In osteoarthritis, SIRT7 levels in cartilage are reduced, which accelerates the catabolism of collagen II by degenerated chondrocytes and reduces their autophagy (a mechanism that has been shown to help improve the phenotype) [146].

## Drugs targeting SIRT7

Because the modulation of sirtuins could have beneficial health effects, there is growing interest in discovering small molecules that modify their activities. In this way, there are currently several sirtuin activators such as resveratrol, SRT1720, UBCS039, oxazolo[4,5-b]pyridine derivatives, imidazo[1,2-b]thiazole derivatives and 1,4-dihydropyridine derivatives [147, 148], as well as inhibitors such as nicotinamide, sirtinol, splitomycin, HR73, AGK2, cambinol, salermide, tenovin, and suramin [147]. However, there is currently no commercially available specific activator or inhibitor for SIRT7. The specific design and development of activators and inhibitors of SIRT7 activity could be beneficial both to better understand the functions of this protein and for its possible application in the treatment of diseases, including those associated with aging. The first attempt to obtain a specific inhibitor of SIRT7 activity provided two cyclic tripeptides whose central residue were inhibitory warheads of the thiourea and carboxamide type. These compounds were able to inhibit tRNA-activated SIRT7 deacetylase activity, but were not as specific as expected [149]. Then, a study using structure-based virtual screening identified two compounds, designated 2800Z and 40569Z, with the ability to specifically inhibit SIRT7 and induce apoptosis and sensitize liver cancer cells in vitro. It is highlighted that R120, W126, and H187 were the critical sites responsible for the interaction of SIRT7 with these small molecules [150]. Another study using an in vitro enzyme activity assay with a series of compounds obtained from a chemical library identified compound ID: 97491 that was shown to induce apoptosis and inhibit sarcoma tumor growth in vivo [151]. Until now, there is no report of the design or development of any specific activator of SIRT7 activity.

## Conclusions and future perspective

As human life expectancy continues to increase around the world, so does the number of people seeking solutions for age-related conditions. Today, 8.5% of people worldwide (617 million) are 65 years of age or older and it is estimated that by 2050 this number will triple, representing almost 17% of the world population (1.6 billion). In this way, and based on everything mentioned above, it is clear that SIRT7 plays an important role in the aging process and its study can contribute to improving people's quality of life. SIRT7 is involved in all the hallmarks of aging, directly or indirectly, and therefore can favor or delay its appearance. In general, it preserves genomic and telomere stability, regulates proteostasis and mitochondrial function, maintains glucose homeostasis, prevents stem cell exhaustion, and participates in intercellular communication and senescence. Similarly, the imbalance in SIRT7 levels can make us more susceptible or resistant to diseases associated with aging, such as cardiovascular and bone diseases, where its levels are reduced, or obesity and cancer, where they are increased.

Since SIRT7 is the least studied human sirtuin, there are still many challenges and opportunities in this field. Among them, the following can be mentioned: knowing its three-dimensional structure, how its transcription and translation are regulated, all the reactions that it catalyzes in vivo, the proteins with which it interacts, the epigenetic marks that it regulates, the post-translational modifications that regulate its enzymatic activities and degradation, and the processes in which it participates and in what way. In addition to this, its clinical application as a marker of diseases or as a therapeutic target must be analyzed and, focusing on the subject of aging, would be useful to know if there is any therapeutic alternative that can prevent the decrease in SIRT7 activity and if it helps improve the conditions of aging without predisposing to the development of diseases (cancer, obesity). Some possible options that could be considered are exercise, some type of diet (supplemented with NAD+, caloric restriction, low-fat diet), or some medication (natural or synthetic origin). It would also be interesting to investigate and obtain clues from those animals that have a longer life than ours, such as some species of whales, elephants and turtles, and also from those that live longer compared to other animals of similar size such as birds, bats, naked and blind mole rats, among others. The design and development of specific inhibitors and activators of SIRT7 would be very practical for future studies and they could also be commercially exploited in the treatment of diseases by complying with the necessary regulations.

In addition to conventional methods, models (cell lines, rodents, flies, and worms) and analyses (qPCR, western blot, ChIP), other more advanced options could be explored. Organoids can be used to study the effects of SIRT7 down- and upregulation in a tissue- and/or organ-specific manner, and then expand the results with the development of new high-throughput animal models. RNA-seq, ChIP-seq, microarray, epigenomic, proteomic, and metabolomic data should be collected and analyzed together. In this way, the implementation of artificial intelligence systems can help identify the key pathways in which SIRT7 participates and their dynamics during aging or in any of its associated diseases and could be used to devise new treatment strategies.

## Data Availability

This review article did not look at any new data. Only results published in previous studies and identified in the reference list below were used.
